# Synthesis and characterisation of highly branched polyisoprene: exploiting the “Strathclyde route” in anionic polymerisation†

**DOI:** 10.1039/c8ra00884a

**Published:** 2018-03-26

**Authors:** Shehu Habibu, Norazilawati Muhamad Sarih, Azizah Mainal

**Affiliations:** Polymer Research Laboratory, Department of Chemistry, Faculty of Science, University of Malaya 50603 Kuala Lumpur Malaysia nmsarih@um.edu.my; Department of Chemistry, Faculty of Science, Federal University Dutse PMB 7651 Jigawa State Nigeria

## Abstract

This work aimed at developing a synthetic route towards highly branched poly(isoprene) from commercially available raw materials, in good yield and devoid of microgelation, *i.e.*, to prepare a completely soluble polymer *via* the versatile technique anionic polymerisation. The polymerisations were conducted under high vacuum conditions using *sec*-butyllithium as initiator at 50 °C in toluene. Toluene served both as a solvent and as a chain-transfer agent. The polar modifier used was tetramethylethylenediamine (TMEDA), and a commercial mixture of divinylbenzene (DVB) was employed as the branching agent for the “living” poly(isoprenyl)lithium anions. The nature of the reaction was studied on the TMEDA/Li ratio as well as the DVB/Li ratio. The obtained branched polymers were characterised by triple detection size exclusion chromatography (SEC), proton nuclear magnetic resonance spectroscopy (^1^H NMR), differential scanning calorimetry (DSC) and melt rheology. Broad molecular weight distributions have been obtained for the highly branched polymer products. ^1^H NMR spectroscopy reveals the dominance of 3,4-polyisoprene microstructure. It was found that the complex viscosities and dynamic moduli of the branched samples were much lower compared to their linear counterparts. The results conform with earlier findings by the “Strathclyde team” for radical polymerisation systems. This methodology has the potential of providing soluble branched vinyl polymers at low cost using the readily available raw materials.

## Abstract 2

Copolymerisation of isoprene and divinylbenzene was achieved in toluene *via* anionic chain transfer polymerization to yield highly branched polymers with good solution and rheological properties.

## Graphical abstract

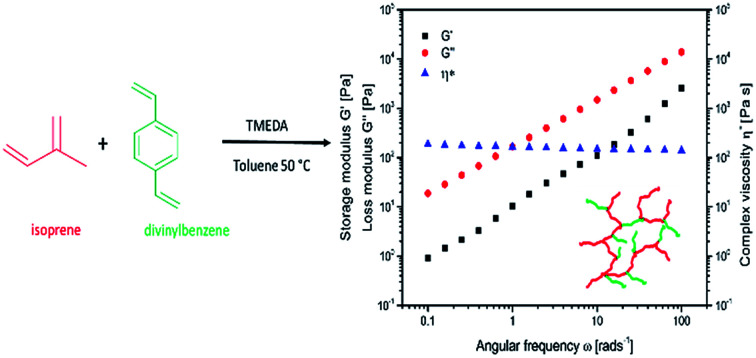

## Introduction

Highly branched polymers are among the most important class of synthetic polymers which have received constant attention from both industry and academia due to their unique chemical and physical properties.^1–3^ Because of their distinctive and favourable properties such as increased solubility, solution and melt viscosities, as well as functional group density, branched polymers have found many potential applications in nanotechnology, catalysis and biomaterial fields.^2,4^ Apart from being good candidates for liquid coating industries due to their improved solubility and lower viscosity,^5,6^ branched and hyperbranched polymers have received much attention over the past decades due to their attractive features such as three-dimensional structures, and end-functionalised groups.^7–9^ Highly branched (hyperbranched) polymers emerged to solve the problems of high cost and synthetic difficulties associated with dendrimers. Unlike dendrimers which are synthesised in multi-step approach, highly branched polymers are prepared in one-pot synthesis, making their potential applications in a large scale, commercially more viable.^3,10^ There are quite some approaches to the synthesis of highly branched polymers, such as polycondensation,^11,12^ self-condensing vinyl polymerisation (SCVP),^13–16^ high-temperature polymerisation,^6^ and free-radical polymerisation technique.^17^ These techniques have been used to synthesise branched polymers of various types including graft polymers, star polymers and miktoarm star-shaped polymers. While the polycondensation, self-condensing vinyl polymerisation and high-temperature methods are not cost-effective, the classical free radical copolymerisation in the presence of an even small amount of multifunctional comonomer leads quickly to the formation of insoluble gels.^6,18^ Thus, preparation of highly branched polymers in good yield, and without gelation remains a challenge to both industrial and academic polymer chemists and engineers. Various synthetic routes have been suggested. A straightforward and cost-effective approach to the synthesis of branched polymers *via* free radical polymerisation was reported by Sherrington and his coworkers, a method known as “Strathclyde methodology”. Strathclyde approach employs the use of appropriate level chain-transfer agents (thiols) to prevent gel formation during the free radical polymerisation of vinyl monomers with difunctional comonomer.^19–24^ The addition of a difunctional comonomer results in chain branching but at the same time could also cause crosslinking and gel formation. However, the incorporation of a chain transfer step into this route causes the termination of the growing polymeric chains as well as the initiation of a new chain and consequently leads to the reduction in the molecular weight and hence, aids in overcoming the problem of gel formation.

Radical polymerisation involving chain transfer to monomer has also been reported as a mean of producing highly branched polymers.^5,25,26^ The use of functionalized 1,1-diphenylethylene (DPE) derivatives in combination with anionic polymerisation has been previously reported as one of the methods to synthesise highly branched polymers.^27^ For over six decades since the proof of its ‘living’ nature demonstrated by Szwarc *et al.*,^28,29^ anionic polymerisation remains the yardstick for measuring other living/controlled polymerisations. This superiority is due to the ability to synthesise well-defined structures of various architectures ranging from linear polymers to dendrimers with controlled molecular weights and several molecular weight distributions, which attracted the scientific community for years. However, the absence of natural death does not mean immortality either.^29^ Hence, the anionic polymerisation technique could be explored in the preparation of highly branched polymers *via* chain transfer to solvent as an analogous to Strathclyde's approach.

This work was therefore aimed at synthesising highly branched poly(isoprene) from commercially available raw materials, in good yield and devoid of microgelation. Copolymerisation of isoprene and divinylbenzene (DVB) was carried out using *sec*-butyl lithium as the initiator, TMEDA was added as the polar modifier, and gelation was prevented by chain transfer to solvent. Some experimental parameters were investigated such as the TMEDA/initiator ratio, as well as the proportion of the branching comonomer to the initiator. The prepared linear and branched polymers were sufficiently characterised. Their thermal and rheological properties were assessed, and a comparison study has been made of the highly branched polymers and the linear polymers samples.

## Experimental

### Materials

Isoprene (99%), *sec*-butyl lithium (1.4 M in cyclohexane), *n*-butyllithium (2.0 M in cyclohexane), and *N*,*N*,*N*,*N*-tetramethylenediamine (99.5%) were obtained from Sigma-Aldrich. Dried methanol (99.9%) and divinylbenzene (98%, mixture of isomers), were purchased from Merck (Germany). Butylated hydroxytoluene (BHT), 99% was obtained from Fischer Scientific. Toluene (HPLC grade), benzene (99.9%), isoprene and divinylbenzene were dried and degassed by several freeze–pump–thaw cycles over calcium hydride (CaH_2_) (Aldrich), on a vacuum line. A customised reactor, “Christmas tree” reaction vessel was used to carry out all the syntheses. The reaction vessel was washed thoroughly with solvents and evacuated overnight followed by rinsing with living poly(styryl)lithium solution to react with and remove any trace amount of impurities remaining in the vessel. The apparatus was further evacuated before the polymerisation.

### Measurements

^1^H NMR analysis was carried out on a DELTA2 (JEOL)-400 MHz spectrometer using CDCl_3_ as the solvent. Spectra were referenced to the traces of CHCl_3_ (7.26 ppm) present in the CDCl_3_. Molecular weights and polydispersity indexes were determined using triple detection size exclusion chromatography (SEC) on a Viscotek 302 with refractive index, viscosity and light scattering detectors, and 2 × 300 mm PLgel 5 μm mixed C columns. THF was used as the eluent with a flow rate of 0.8 ml min^−1^ and at a constant temperature of 35 °C. The detectors were calibrated with a single poly(styrene) standard obtained from Polymer Laboratories, and values of d*n*/d*c* (ml g^−1^) of 0.127 and 0.087 was used for linear poly(isoprene) and branched poly(isoprene) respectively.

### Thermal analysis

Thermal properties of the branched polymers were investigated by differential scanning calorimetry (DSC). Phase transition temperature was identified with a DSC 822e, Mettler Toledo calorimeter equipped with Haake EK90/MT intercooler. The calorimeter was calibrated using standard indium for temperature and enthalpy accuracy before experiments. All the samples tested were dried in a vacuum oven over di-phosphorous pentoxide for at least 48 hours. About 4–8 mg of each sample was heated under nitrogen atmosphere at a scanning rate of 10 °C min^−1^ after the material was being encapsulated in the aluminium pans. The range for the measurement was from −40 to 200 °C and the samples were first heated from room temperature to 200 °C, and then cooled to −40 °C. The second heating cycle was performed in the range −40 °C to 200 °C and data from this cycle was used for the analysis. The data were analysed using STARe Thermal Analysis System software.

### Melt rheology

The linear oscillatory measurement was performed on an Anton Paar MCR301 rheometer using a 25 mm parallel plate geometry with a gap of 1 mm and a convection temperature device (CTD). Amplitude sweep experiments were initially conducted to determine the linear viscoelastic regime. Frequency sweep measurements measured dynamic moduli for frequencies from 0.1 rad s^−1^ to 100 rad s^−1^ in the linear viscoelastic regime. Measurements were conducted over a temperature range 40 to 120 °C, at 10° intervals for all samples. All the samples were stabilised with antioxidant (BHT) and vacuum dried to prevent oxidative degradation. The storage modulus, loss modulus as well as complex viscosity were evaluated.

### Synthesis of linear and branched polyisoprene

All polymerisations were carried out by living anionic polymerisation using standard high vacuum techniques similar to the one previously reported.^30^

#### Linear poly(isoprene)

A typical procedure was as follows; toluene (100 ml) was distilled into the reaction vessel, followed by 5.2 g (0.0763 mol) of isoprene with the aid of an attachable flask. 0.1 ml (0.20 mol) of *n*-butyllithium was injected into the monomer in the attachable flask before being transferred to the reaction vessel. After the distillation of solvent and monomer, 0.22 ml (1.47 mol) of TMEDA (2 molar equivalent) was injected into the reaction followed by 0.52 ml (0.728 mol) of *sec*-butyllithium to initiate the reaction as presented in Table 1.

**Table 1:** Synthesis and molecular characterisation of linear polyisoprene

Sample	TMEDA/Li	*M*_n_ (g mol^−1^)	*Đ*	Yield (%)	Microstructure	
					1,4 (%)	3,4 (%)
L1	0	19 100	1.04	91	92	08
L2	0.5	11 300	2.1	92	11	79
L3	1	9300	2.25	75	21	55
L4	0.5	57 500	1.88	84	12	78
L5	1.5	7300	3.05	94	12	71
L6	2.0	9700	5.04	72	09	67

The reaction was maintained at 50 °C and was allowed to stir overnight. (0.1 ml, 2.47 mol) N_2_-sparged methanol was used to terminate the reaction and produce homopolymers of isoprene which was collected by precipitation with excess methanol. BHT stabiliser was added after termination as well as before the precipitation to prevent oxidation. ^1^H NMR (400 MHz; CDCl_3_; Me_4_Si) *δ*_H_ 1.32 (br, 3H, s, 1,4-, aliphatic); 1.70 (br, 3H, s, 1,4-) 1.80 (br, 2H, m); 2.03 (4H, br, 1,4-, aliphatic); 2.26 (1H, br, 3,4-, aliphatic); 4.68 (1H, br, olefinic); 4.72 (1H, br, 3,4, olefinic); 5.12 (1H, s, 1,4-, olefinic). A similar procedure was adopted to prepare the remaining polymers by varying the TMEDA/Li from 0.5 to 2.0.

#### Branched poly(isoprene)

**Scheme 1:**
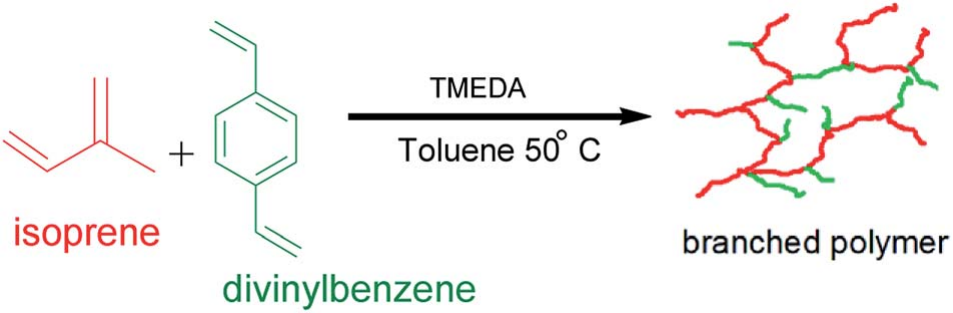
Synthesis of branched poly(isoprene) *via* anionic copolymerisation of isoprene with DVB.

A typical procedure was as follows; 100 ml of toluene was distilled into the reaction vessel followed by 7.43 g (0.1091 mol) of isoprene with the aid of an attachable flask. 0.1 ml (0.20 mol) of *n*-butyllithium was injected into the monomer in the attachable flask before being transferred to the reaction vessel. After the distillation of solvent and monomer, 0.06 ml (0.4008 mol) (0.5 molar equivalent) TMEDA was injected into the reaction followed by 0.531 ml (0.743 mol) of *sec*-butyllithium to initiate the reaction. 0.22 ml (1.5071 mol) of divinylbenzene was injected 5 minutes after initiation, the reaction was maintained at 50 °C and allowed to stir overnight (Scheme 1). The polymerisation was terminated with (0.1 ml, 2.47 mol) N_2_ sparged methanol and recovered by precipitation in excess methanol containing a small amount of BHT. ^1^H NMR (400 MHz; CDCl_3_; Me_4_Si) *δ*_H_ 1.79–2.06 (14H, m, 1,4, aliphatic), 2.26 (1H, s, 3,4-, aliphatic), 4.64 (7H, br. s., 3,4), 4.67–4.76 (5H, m, 3,4), 4.77–4.94 (3H, m, 1,2), 4.94–5.12 (3H, m, 1,4-, olefinic),7.13–7.18 (3H, m, Ar). A similar procedure was adopted for all other branched polymer samples by varying the TMEDA/Li and the DVB/Li from 0.5 to 2.0 and 1.0 to 4.2 respectively.

## Results and discussion

**Scheme 2:**
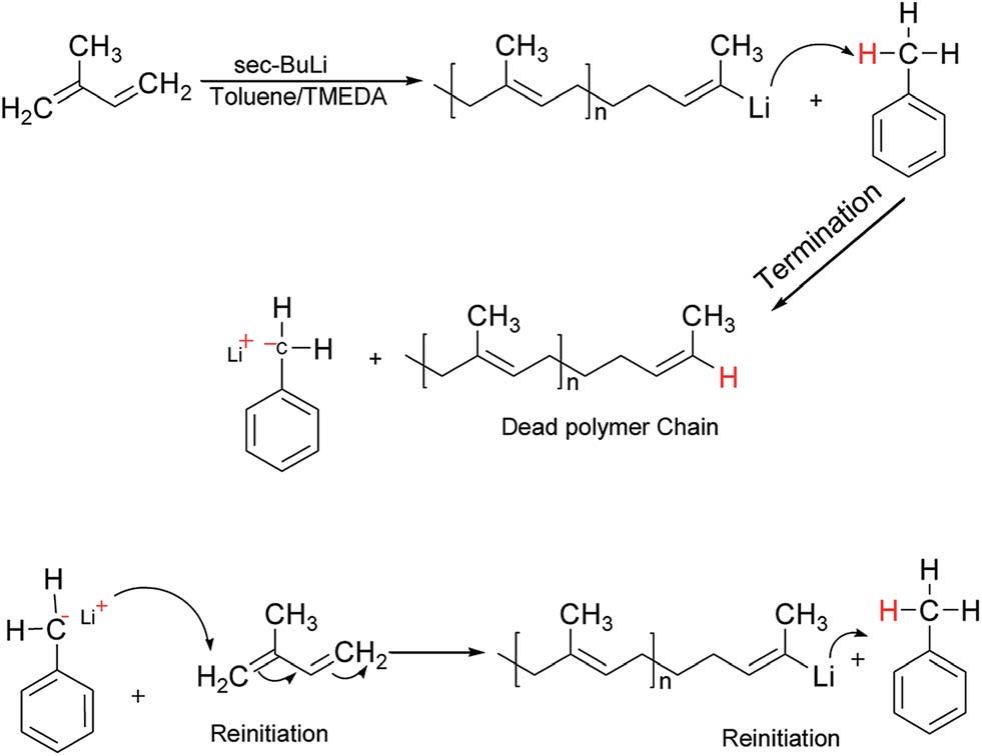
Mechanism of chain transfer to toluene in anionic polymerisation of isoprene.

It is well-known that the type of solvent used strongly affects the anionic polymerisation of diene monomers such as butadiene and isoprene. Moreover, the addition of Lewis bases such as TMEDA in a system containing lithium as a counter ion results in the formation of uncontrolled polymeric product with high dispersity index due to chain transfer reaction under appropriate conditions.^31^ For example, when butadiene was polymerised in toluene at 40 °C in the presence of TMEDA, polymer with broad molecular weight and low 1,4-microstructure were obtained.^32,33^ However, due to the scarcity of reliable information on the effectiveness of the chain transfer process, the behavior of isoprene in toluene was initially investigated without the addition of the branching comonomer. Six linear samples (L1–L6) with varying amount of polar modifier were prepared (Table 1). These polymer samples were obtained in good yield (>85%), and there was a remarkable decrease in the molecular weight as well as an increase in the dispersity index as the level of TMEDA/Li increases. The anionic polymerisation of isoprene in toluene proceeds with chain transfer to solvent as depicted in Scheme 2, and the presence Lewis base, *N*,*N*,*N*′,*N*′-tetramethylethylenediamine enhanced the chain transfer.^32^

**Fig. 1:**
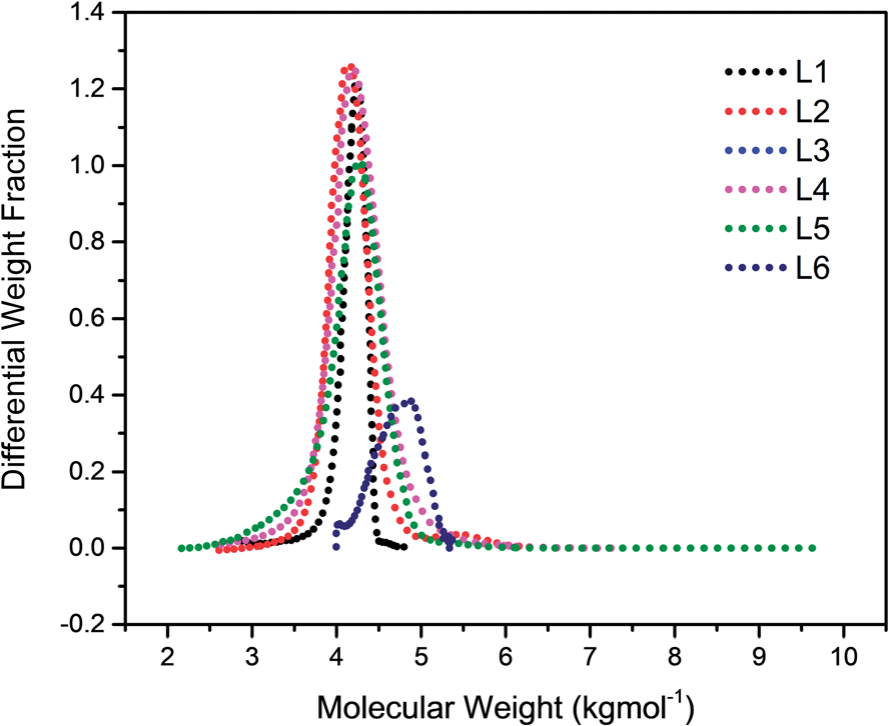
Molecular weight distribution curves for linear polymers prepared with varying amounts of TMEDA/Li: L1 (0); L2 (0.5); L3 (1); L4 (0.5); L5 (1.5); L6 (2).

The chain transfer process takes place in two stages, termination of the growing polymer chain followed by re-initiation. Since the point at which gelation occurs depend upon the polymer chain length as well as the crosslink density, in the absence of the termination, the polymerisation would proceed with gel formation. Therefore, the termination stage of the chain transfer process serves to reduce the polymer chain length before it reaches the point of gelation. Fig. 1 presents the molecular weight distribution curves for the linear samples, L1–L6. The curves were remarkably similar in their distribution and broadened with an increase in the amount of TMEDA. Although there is no apparent systematic trend in the molar mass distribution of these linear polymers, the level of TMEDA used has an influence on the molar mass distributions. The branched polymers were synthesised with varying ratios of TMEDA/Li to understand the effect of the chain transfer process.

The TMEDA/Li was varied from 0.5 to 2.0 to promote the chain transfer and obtain a favourable 1,4-microstructure of the resulting branched polymer.

### Synthesis of hyperbranched polymer

**Scheme 3:**
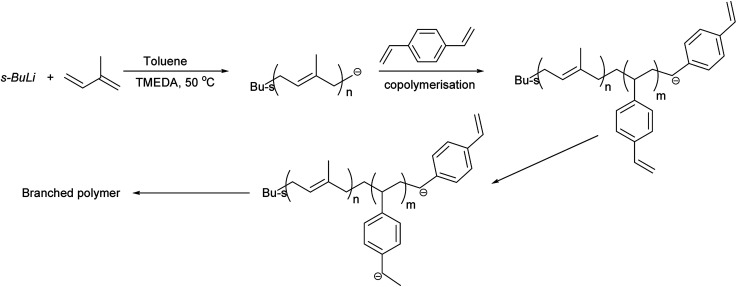
Proposed mechanism of formation of highly branched polyisoprene.

Copolymerisation of isoprene and divinylbenzene was carried out to produce branched polyisoprene. Polymers were obtained in good yield (>85%), and various ratios of TMEDA were employed for different DVB/Li ratios. The results of copolymerisation of isoprene and divinylbenzene are summarised in Table 2 and the proposed branching mechanism is presented on Scheme 3.

**Table 2:** Synthesis and molecular characterisation of branched polyisoprene

Sample	DVB : Li	TMEDA : Li	*M*_n_ (g mol^−1^)	*Đ*	Yield (%)	[*η*]_hyper_ (dl g^−1^)^a^	PI-Microstructure		[*η*]_linear_ (dl g^−1^)^b^	*g*′^c^	*T*_g_ d
							1,4 (%)	3,4 (%)			
B1	1.0	1.0	86 800	2.49	98	0.5324	14	57	1.477	0.36	−14.85
B2	1.0	1.5	54 500	1.37	97	0.2802	17	73	0.658	0.43	−15.62
B3	1.0	0.5	17 900	2.04	89	0.2404	18	72	0.400	0.60	−19.53
B4	1.2	1.2	21 900	1.54	85	0.377	08	70	0.4272	0.88	−16.57
B5	1.2	1.5	34 800	6.51	82	0.4802	11	60	1.527	0.31	−12.06
B6	1.2	1.0	19 800	1.99	88	0.1896	09	81	0.424	0.45	−16.12
B7	2.0	1.5	17 500	3.17	78	0.313	14	76	0.542	0.58	−24.41
B8	2.0	0.5	79 600	3.88	85	0.724	16	77	1.917	0.38	−27.17
B9	3.0	1.0	141 600	2.19	98	0.2871	15	67	1.926	0.15	−9.52
B10	3.0	1.5	16 900	6.39	86	0.3151	12	70	0.885	0.36	−23.28
B11^e^	3.8	2.0	—	—	∼100	—	—	—	—	—	
B12^e^	4.2	0.8	—	—	∼100	—	—	—	—	—	

a Measured by SEC in THF at 35 °C.

b Calculated using Mark–Houwink–Sakurada equation: [*η*]_lin_ = *KM*_w_^*α*^; *K* = 0.000177 dl g^−1^, *α* = 0.735 dl g^−1^.

c *g*′ = [*η*]_hyper_/[*η*]_lin_.

d Measured by DSC.

e Formed an insoluble gel.

### Triple detection size exclusion chromatography

**Fig. 2:**
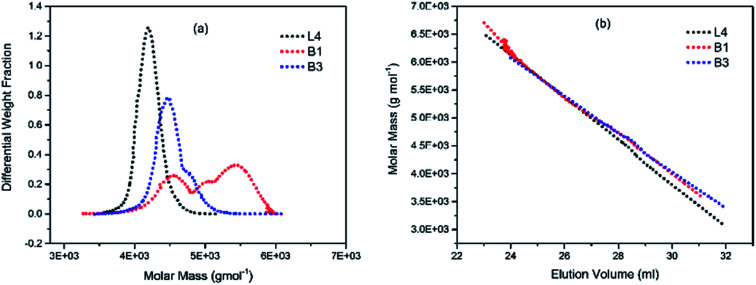
(a) Molar weight distribution curves; and (b) molecular weight–elution volume plots for L4 (no DVB), B1 and B3 (DVB/Li = 1).

**Fig. 3:**
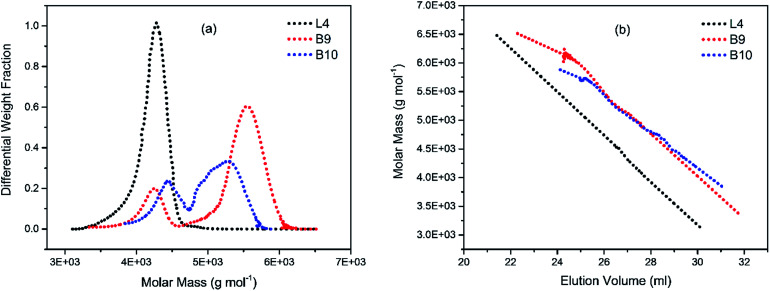
(a) Molecular weight distribution curves; and (b) molecular weight–elution volume plots for L4 (no DVB), B9 and B10 (DVB/Li = 3).

**Fig. 4:**
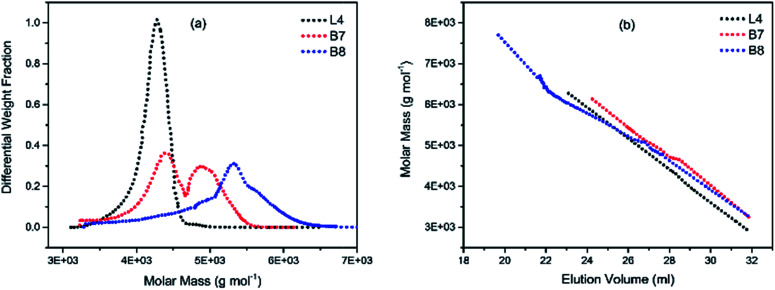
(a) Molecular weight distribution curves; and (b) molecular weight–elution volume plots for L4 (no DVB), B7 and B8 (DVB/Li = 2).

**Fig. 5:**
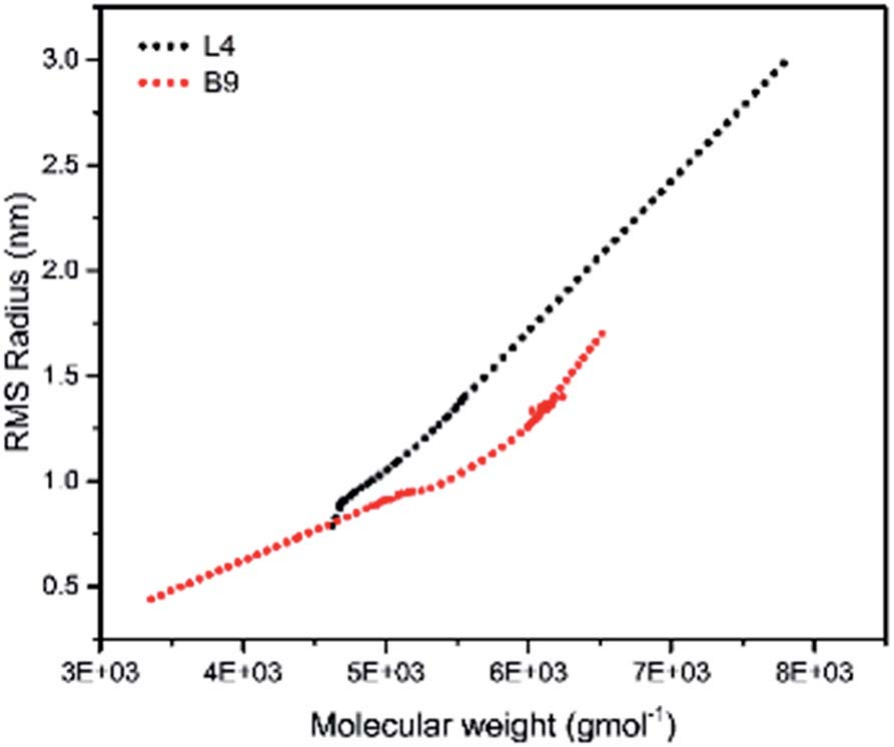
Root mean square radii of gyration *versus* molar mass for L4 and B9.

To illustrate the branching nature of the branched polymers (Table 2), their elution properties were compared with those of a linear sample L4 which was prepared with a TMEDA/Li ratio of 0.5 and *M*_w_ of 108 200 g mol^−1^. Various amounts of TMEDA were used to prevent gelation in the synthesis of the branched polymers. All the branched polymers possess higher values of *M*_w_ than those of the linear ones. It is evident from Fig. 2–4 that the molar mass distributions (*Đ*) of the branched samples were broader when compared to the linear samples. Moreover, there were manifestations of multimodal spreading, and this is consistent with polymer samples having a randomly branched architecture.^19,23,34–38^ It is clear that these branched polyisoprenes are complex both regarding molar mass and architectural distributions. It is evident from Fig. 2(a) (DVB/Li = 1), 3(a) (DVB/Li = 2) and 4(a) (DVB/Li = 3) that the branched samples possess broader distributions compared to the linear polymer. The molecular weight *versus* elution volume plots in Fig. 2(b), 3(b), and 4(b) all showed the branched samples at the upper right with respect to linear counterparts; this signifies the branched nature of these polymers once more. Similar observations were reported by other researchers.^19,23,35–38^ Furthermore, upon increasing the DVB ratio from 1 to 3, the gap between the linear and the branched samples on the molecular weight *vs.* elution volume plot becomes wider to indicate higher levels of branching.^19,37^ The contraction factor, *g* which is the ratio of the mean square radius of gyration of the branched sample to that of the linear sample of the same molecular weight is one of the standard measures of polymer branching.^2,39^ Since branched polymers are expected to be more compact than their linear counterparts of the same molecular weights, the *g* value is unity for the linear polymers, and decreases with the increase in the degree of branching. The root-mean-square radius (RMS) of gyration could be measured by SEC coupled with light scattering detector. However, low molecular weight polymers have weak scattering, and it is, therefore, challenging to obtain a useful data for polymers with RMS radii ≤ 10–15 nm as reported by other researchers.^37,40^ This, indeed a constraint, applies to most of the polymers synthesised in the present study. However, at appropriately high molecular weight some information could be derived. Comparing the molecular weights and RMS radii data of the linear sample L4 and the branched B9 having the highest molecular weights in their particular sets (Fig. 5), reveals that the linear sample has larger radii than the branched polymer sample at any given molecular weight slice. A similar observation was reported by others.^36,37^ A closer look at the polymerisation composition reveals the formation of soluble branched polymers with up to DVB/Li ratios of up to 3.0 that is, three units of DVB molecules for every initiator fragment. Attempt to synthesise branched polymer with a DVB ratio of 3.8 resulted in the formation of cross-linked polymer even when the TMEDA/Li ratio was 2.0. Usually, vinyl copolymerisation involving divinyl benzene proceeds with crosslinking and gel formation even at an early stage of the polymerisation. However, under appropriate conditions, soluble branched polymers could be obtained in the anionic polymerisations involving divinylbenzene or another multifunctional comonomer.^41^ The solubility of the polymer formed is attributed to the fact that pendant vinyl groups in the polymer chain are much lower in reactivity than the vinyl group of the divinylbenzene. The chain transfer reaction may precede the pendant vinyl group attack by the chain end anion which would otherwise lead to crosslinking and gel formation. Dissimilarity in reactivity between the vinyl groups in divinylbenzene and other monomers was reported by a kinetic study of the model compounds.^41,42^ However, crosslinking may occur at the final stage of the polymerisation, resulting in the formation of an insoluble gel. Formation of soluble polymers in anionic polymerisation involving divinylbenzene and lithium diisopropylamine was reported,^43^ and it was suggested that the excess diisopropylamine would stabilise the chain-end carbanion thereby rendering it inactive towards the pendant double bonds. Similarly, chain end anions may be rendered less reactive in the presence of TMEDA due to strong coordination between the Li^+^ and TMEDA molecules thereby shifting the carbanionic chain end bearing the Li^+^ to a highly reactive solvent-separated ion pair. Consequently, the system would be changed to non-living and the molecular weight of the resulting polymer would be different from the predicted by the ratio of the monomer-initiator.^1^

### Effect of TMEDA on polyisoprene microstructure

Polyisoprene produced by anionic polymerisation using Li initiator has a high 1,4-content (>90%) analogous to natural rubber. That leads to the growth of commercial importance of anionic polymerisation.^44^ However, the addition of polar solvents especially THF to hydrocarbon media can drastically alter the microstructure from the high 1,4- to mainly 3,4- and some 1,2- units. Similarly, the addition of Lewis bases such as TMEDA, even in small amounts can significantly alter the microstructure.^45^ Four different combinations of TMEDA/initiator ratios were used in this study. All polymerisations were achieved at a constant temperature of 50 °C; this ruled out the effect of temperature on the microstructure of polyisoprene.^46,47^ The content of isoprene isomeric units in the polymers synthesised was determined using ^1^H NMR data (Table 2). According to the literature, the characteristic peaks of the alkene protons of the polyisoprene components exist in the range of 4.5–5.5 ppm. The peaks corresponding to 1,4-microstructure are visible at *δ* 5–5.2 ppm [(CH_3_)C=CH] and those corresponding to 3,4-microstructure are found at *δ* 4.6–4.8 ppm [(CH_3_)C=CH_2_].^42,45^ It was observed that the addition of TMEDA resulted in a shift in the microstructure from the mainly 1,4-microstructure to pre-dominantly 3,4-microstructure.

### Differential scanning calorimetry (DSC)

DSC was the technique employed to determine the glass transition temperature (*T*_g_) of the polymers. *T*_g_ provides information about the mobility or rigidity of polymers. *T*_g_ also provides a temperature range of practical application, processing conditions as well as identification and comparison. The *T*_g_ values for the branched polymers were listed in Table 2 and ranged from −27.74 to −9.52. The least values were obtained for the DVB–Li ratio of 2. The glass transition temperature is known to be affected by several factors that are related to the chemical structure of polymers. These factors include the molecular weight, the flexibility of the backbone, the molecular structure, as well as the existence of branching in a polymer.^48^ In the case of polyisoprene, the microstructure also seems to play a significant role, and therefore different microstructures result in different *T*_g_ values.^49^ Living anionic polymerisation of isoprene in non-polar solvents using lithium initiator would be expected to produce polyisoprene with over 70% 1,4 microstructure with *T*_g_ of about −73.^32,49–51^ However, in the present study, 3,4-microstructure dominates due to the presence of TMEDA and this 3,4-polyisoprene will cause an increase in the *T*_g_ of the resulting polymer. Moreover, the mobility of the polymer backbone has a significant influence on the *T*_g_ of polymers, and this is primarily determined by the chemical composition and spatial structure. For example, the presence of aromatic rings hinders the free rotation of the polymeric chains. Hence, more thermal energy is required for chain mobility, and *T*_g_ increases. For highly branched polymers, several co-operative interactions are responsible for the variation of glass transition temperature. As the degree of branching increased, the mobility of the molecular chain decreases due to the increase in compactness of the spatial distribution of the molecular structure. The increase in the degree of branching, at the same time, can cause an increase in the molecular mobility due to an increase in the free volume of the molecular chains.^52–54^ As can be seen in Table 2, samples B7, B8 and B10 having the lowest *g*′ possess the lowest *T*_g_ values. Sample B9, on the other hand, has the highest *T*_g_ values possibly due to the high molecular weight as well as high DVB content.

### Melt rheological characteristics

**Fig. 6:**
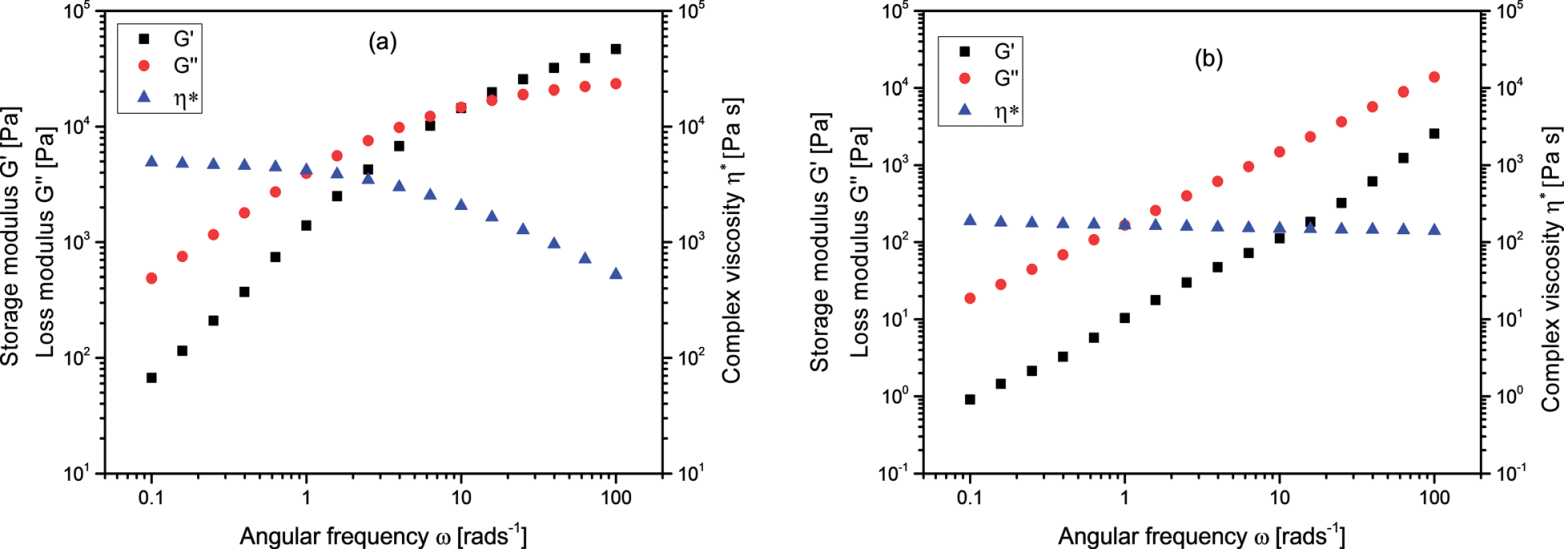
Complex viscosity (η*) storage modulus *G*′, and loss modulus *G*′′, for (a) linear, L4 and (b) branched, B10 polymers at 70 °C.

In addition to their solution properties, the melt rheological behavior of branched polymers is one of their most essential features. Melt rheological properties of polymers are known to be influenced by three important molecular structural parameters for instance; molecular architecture (degree of branching), molecular weight as well as molecular weight distribution.^37,54–56^ Linear oscillatory experiments were performed in an Anton Paar MCR 301 rheometer, and there was a strong relation between the melt rheological properties and the degree of branching of the branched polymers. Fig. 6 shows the complex viscosity, η*, storage modulus, *G*′, and loss modulus *G*′′, for (a) linear (L4) and (b) branched (B10) polymers respectively. It could be seen that the *y*-axis for L4 almost covers the range from 10^1^ to 10^5^ Pa whereas for B10 it ranged from 10^−1^ and extended to slightly above 10^4^ Pa. There is a decrease in the complex viscosity at high frequency for both the linear and branched samples which is a typical shear thinning behavior. The shear thinning behavior indicates the pseudoplastic behavior of the melt at this temperature. The complex viscosity, η* for the linear sample L4 was considerably higher than that of the branched sample B10, this is related to the nature of the molecular chains. Fig. 6(b) represents a typical curve for the branched polymers. The storage modulus, *G*′ is a measure of the elasticity of materials, at low frequencies, the *G*′ varies with frequency in approximately quadratic fashion for L4 but almost linearly for the branched sample, B10. However, at high frequency, there is a slight elastic rubber plateau in the case of linear sample L4 due to polymer chain entanglements.^57,58^ Meanwhile the loss modulus, *G*′′ reveals the viscous nature of the material which dominates and varies linearly with frequency for both the linear and branched samples. The *G*′′ indicates the energy lost to the viscous deformation in the course of the deformation of materials; it reveals the viscosity of materials. The higher the *G*′′ value, the higher the viscosity of the polymer. Poly(isoprene) possess a flexible polymer backbone; it is easy for the linear samples with a long chain to entangle and prevents reorientation of the polymer chain, while the short molecular chains of the very high degree of branching are difficult to entangle. Therefore, there is a less steric hindrance during flow process hence decrease in complex viscosity for the highly branched samples.

**Fig. 7:**
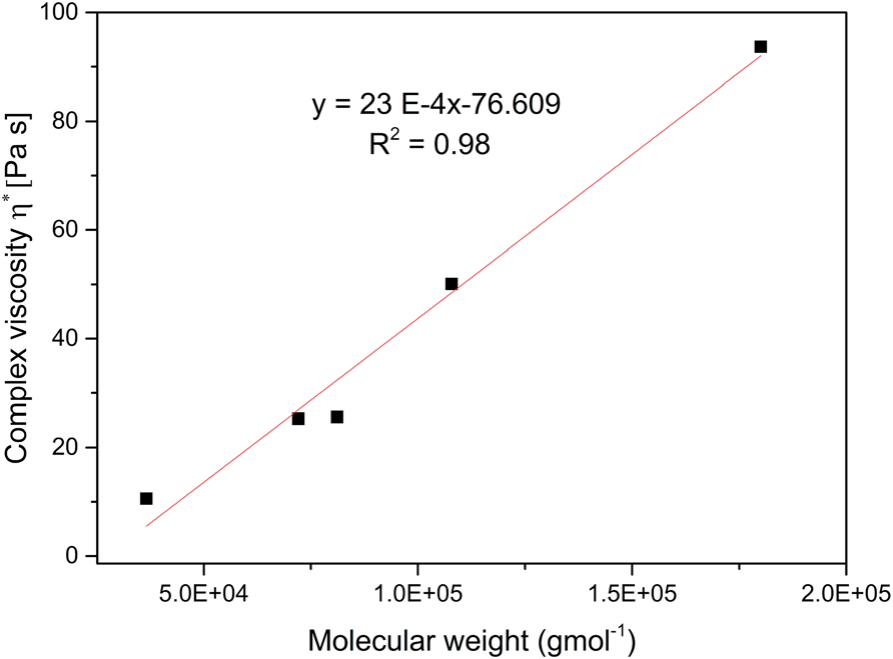
Complex viscosity, η* *versus* molecular weight, *M*_w_ for the branched polymers at 70 °C.

Fig. 7 shows the dependence of shear rate viscosity on molecular weight, *M*_w_. The complex viscosity data at 1 rad s^−1^ for each branched sample were extracted at 70 °C. However, the data do not always represent the zero shear viscosity, *η*_0_ values since they are difficult to obtain experimentally with reasonable accuracy. Nevertheless, it is useful for the purpose of comparison. For linear polymers, the *η*_0_ depends on the *M*_w_ if the polymer is entangled with a slope of about 3.4, as predicted by the classical Mark–Houwink–Sakurada equation: [*η*] = *KM*^*α*^.^37,59^

As the molecular weight of the polymer increases, there is a corresponding increase in internal friction during molecular motion. Also, the thermomechanical movement of the long chain molecules causes the whole molecule to entangle thereby making the flow of the entire molecular chain more difficult at high *M*_w_, consequently η* increases. This entanglement is a typical feature of linear polymers.^37,59^ However, in contrast, the relationship between the *η*_0_ and *M*_w_ for the branched polymers does not follow this equation. With a very low slope of 23 × 10^−4^ indicating the absence of entanglement. This observation has been earlier reported for other hyperbranched polymers and dendrimers.^37,59–61^

## Conclusions

Highly branched polyisoprene has been successfully synthesised with a divinylbenzene in the presence of a chain transfer process in anionic polymerisation to prepare a soluble polymer. The divinylbenzene introduces branching points into the polymer backbone, while the chain transfer mechanism decreases the molecular weight of the polymeric backbone. Since the gel point depends on the crosslink density as well as the chain length, the chain transfer can aid in preventing gelation through chain termination before gelation. This method was a modified approach to the “Strathclyde route” in which a free radical polymerisation was employed. In the present study, anionic polymerisation was used. Since anionic polymerisation allows for the control of molecular weights through the utilisation of an appropriate amount of initiator, chain transfer mechanism can be utilised as an added feature. Higher DVB/Li ratios resulted in crosslinked polymer products. ^1^H NMR spectroscopy analysis reveals the dominance of 3,4-polyisoprene microstructure which increases as the TMEDA/Li increases. Size exclusion chromatography was used to demonstrate the branching nature of the prepared polymers. The branched polymers prepared in this study were compared to their linear counterparts and found to have favourable rheological, and solution properties hence could serve as rheological modifiers.

## Conflicts of interest

There are no conflict of interests to declare.

## Supplementary Material

RA-008-C8RA00884A-s001
