# Supplementary material for: Synthesis and characterisation of highly branched polyisoprene: exploiting the “Strathclyde route” in anionic polymerisation
Source: RSC Adv. 2018 Mar 26;8(21):11684–92. doi: 10.1039/c8ra00884a (PMC9079075; doi:10.1039/c8ra00884a)
Supplement: RA-008-C8RA00884A-s001 [file RA-008-C8RA00884A-s001.pdf]

Electronic Supplementary Material (ESI) for RSC Advances.
This journal is © The Royal Society of Chemistry 2018

Electronic Supporting Information (ESI)

Synthesis and characterization of highly branched polyisoprene: Exploiting the "Strathclyde route" in anionic polymerization

Shehu Habibu,[a,b] Norazilawati Muhamad Sarih[a†] and Azizah Mainal[a]

[a] *Polymer Research Laboratory, Department of Chemistry, Faculty of Science, University of Malaya, 50603 Kuala Lumpur, Malaysia.*

[b] *Department of Chemistry, Faculty of Science, Federal University Dutse, PMB 7651, Jigawa State Nigeria.*

† corresponding author Email: nmsarih@um.edu.my

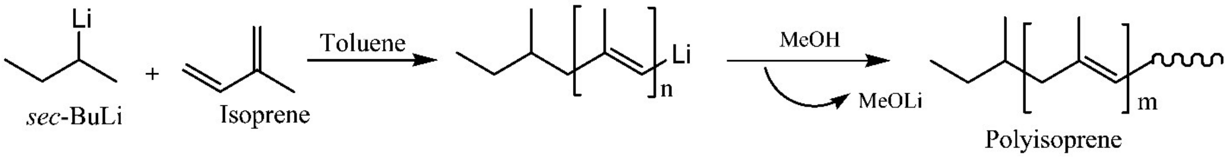

Scheme S1: Synthetic route of homopolymer polyisoprene

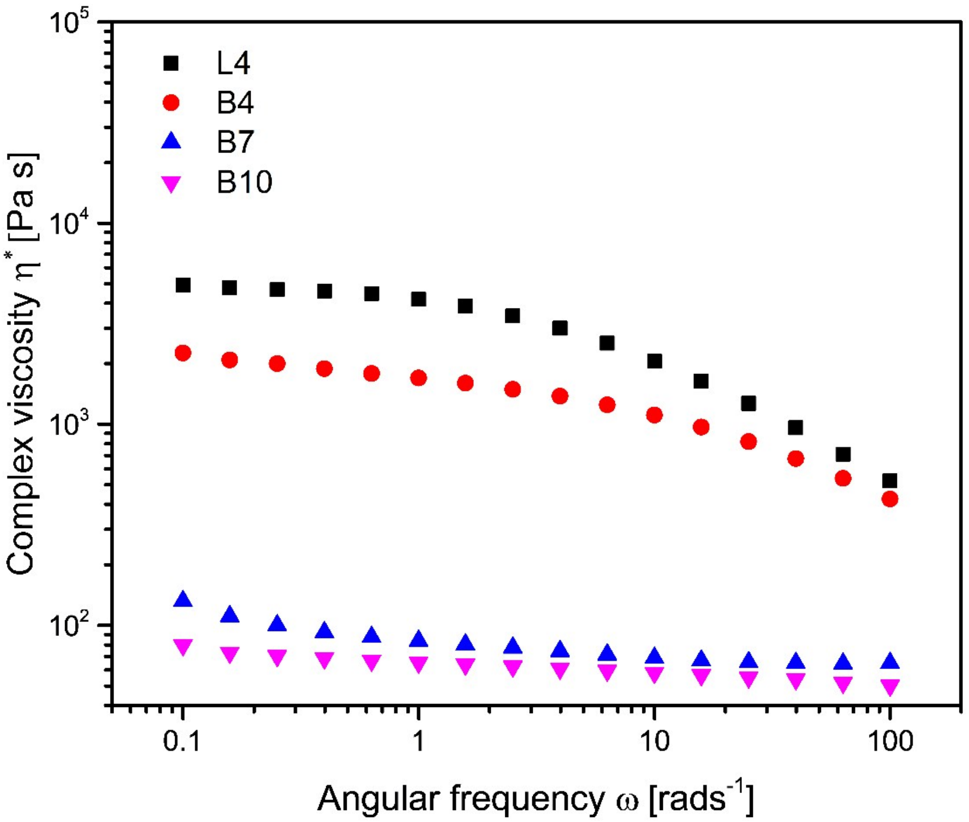

Fig. S1: *Complex viscosity (η,*) for the linear, L4 and branched, B4, B7 and B10 polymers at 70 °C*

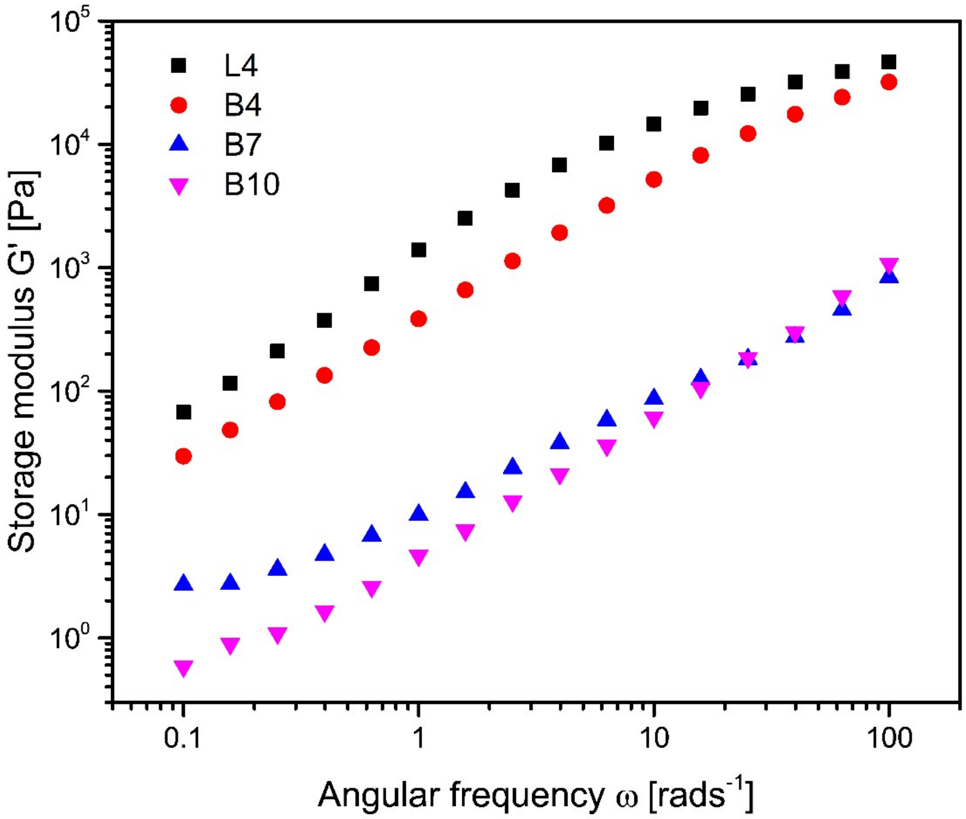

Fig. S2: *Storage modulus G’, for the linear, L4 and branched, B4, B7 and B10 polymers at 70 °C*

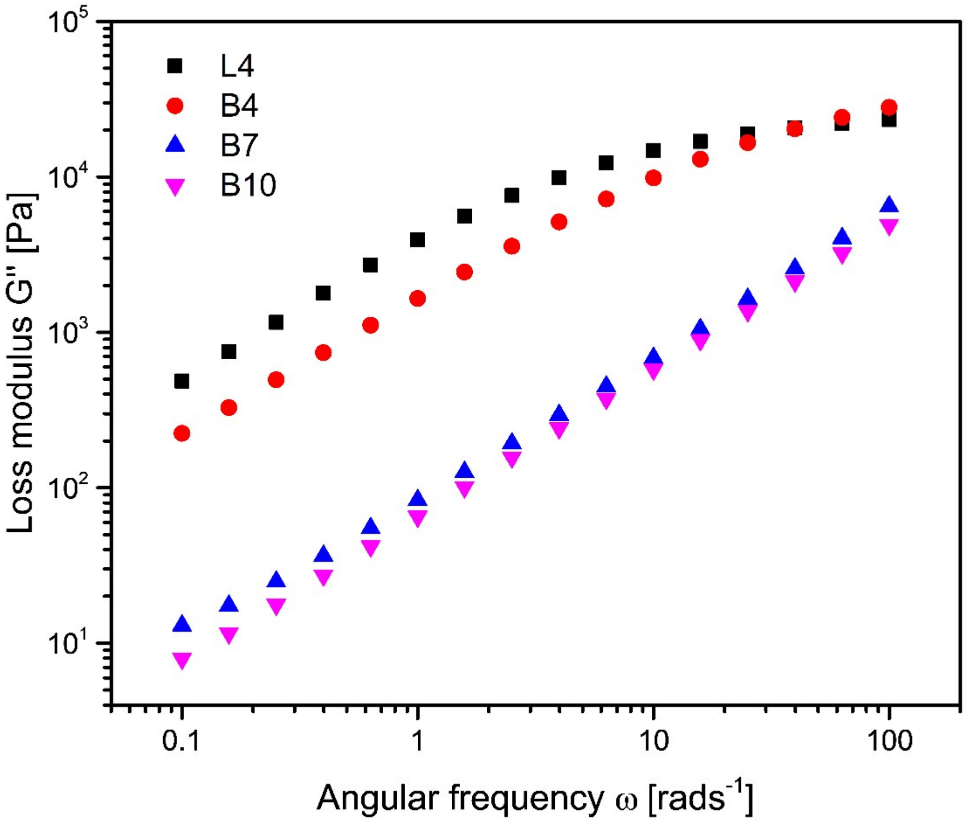

Fig. S3: *Loss modulus (G''), for the linear, L4 and branched, B4, B7 and B10 polymers at 70 °C*

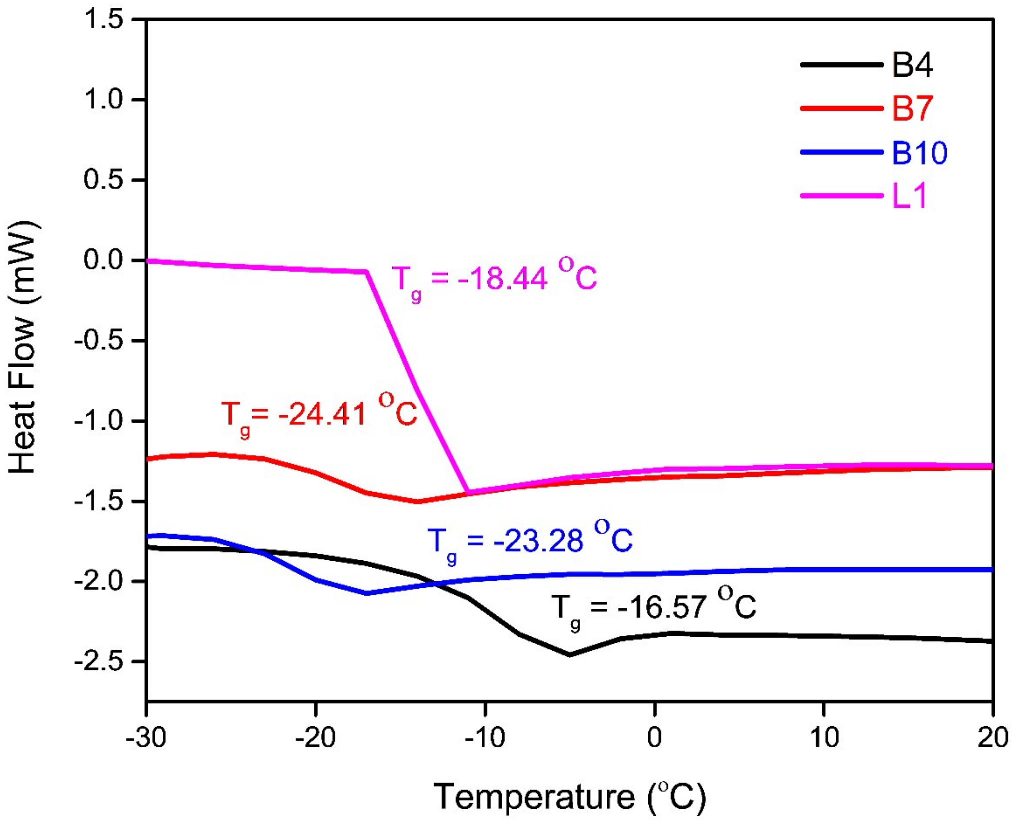

Fig. S4: *DSC thermograms, for (a) linear, L1 and (b) branched, B4, B7 and B10 polymers at 70 °C*

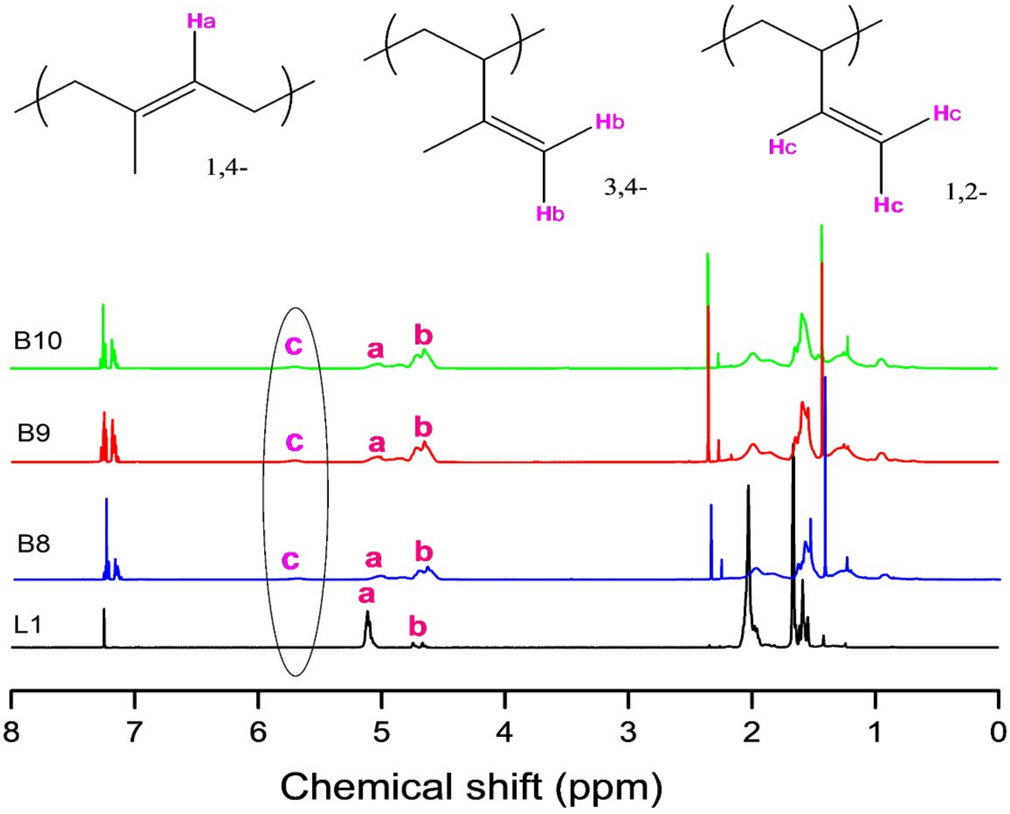

Fig. S5: *^{1}H NMR spectra for linear (L1, TMEDA/Li = 0) and branched (B8, TMEDA/Li = 0.5; B9 TMEDA/Li = 1.0; B10, TMEDA/Li = 1.5) polymers*
